# Supplementary material for: SyNDI: synchronous network data integration framework
Source: BMC Bioinformatics. 2018 Nov 6;19:403. doi: 10.1186/s12859-018-2426-5 (PMC6219086; doi:10.1186/s12859-018-2426-5)
Supplement: Supplementary file 5 — Files of the example presented in Additional file 4. This additional file is a zip package that contains all files used in Additional file 4. Detailed descriptions of each of these files are presented in a README file included in this zip package. (GZ 30411 kb) [file 12859_2018_2426_MOESM5_ESM.gz › SyNDITools/Meme2Fimo/meme_4.9.0_4.tar.gz!/meme_4.9.0/doc/glam2_ref.html]

glam2 Reference


# glam2 Reference

## Usage

```
glam2 [options] alphabet my_seqs.fa
```

An alphabet other than p or n is interpreted as the name of an alphabet file.

## Options that determine which alignments are possible

-o
:   output directory; will not clobber existing files

-O
:   output directory (glam2\_out); allow clobbering

-2
:   Search both strands.

-z
:   Specify the minimum number sequences that must participate in
    the alignment. If greater than the number of input sequences, then all
    the sequences must participate.

-a
:   Specify the minimum number of key positions (aligned columns)
    in the alignment.

-b
:   Specify the maximum number of key positions (aligned columns)
    in the alignment.

## Options that affect the scoring scheme

These options specify parameters in the formula for calculating
alignment scores. See GLAM2 methods (PDF)
for details.

-d
:   Specify a Dirichlet mixture
    file, which describes residues' tendencies to align with one another.

-D
:   Specify the deletion pseudocount.

-E
:   Specify the 'no-deletion' pseudocount.

-I
:   Specify the insertion pseudocount.

-J
:   Specify the 'no-insertion' pseudocount.

-q
:   Specify the weight for generic versus sequence-set-specific
    residue abundances. The residue abundances are estimated by counting
    the residue types in all the input sequences, and adding pseudocounts.
    The total number of pseudocounts is equal to the alphabet size
    mutiplied by the -q parameter. The allocation of pseudocounts among the
    residue types depends on the alphabet.

## Options that affect the search algorithm

glam2 uses a simulated
annealing algorithm, with a temperature parameter. At high temperatures,
glam2 only slightly favours changes that increase the alignment's score,
and at low temperatures it strongly favours such changes. Thus, at high
temperatures the score will be optimized too slowly, but at low
temperatures the algorithm will get frozen in a local optimum. The
strategy, then, is to start with a high temperature and reduce it as
slowly as possible.

-r
:   Specify the number of alignment runs.

-n
:   Specify how many iterations should pass since the
    highest-scoring alignment seen so far before ending each alignment run.

-w
:   Specify the initial number of key positions (aligned columns)
    in the alignment. If less than the minimum (-a) or greater than the
    maximum (-b), it is increased to the minimum or reduced to the maximum.

-t
:   Specify the initial temperature.

-c
:   Specify the cooling factor per n iterations. The temperature
    is multiplied by a constant factor after each iteration, such that
    after n iterations, it has dropped by this amount. 'n' is the number
    specified with the -n option.

-u
:   Specify the minimum temperature. The temperature never drops
    below this level, to avoid numerical problems.

-m
:   Specify the rate of column sampling relative to site sampling.
    On each iteration, glam2 randomly decides to try either realigning a
    sequence or adjusting an aligned column (key position). This parameter
    sets the probabilities for this decision.

-x
:   Specify the site sampling algorithm: 0=FAST, 1=SLOW, 2=FFT.
    See GLAM2 methods (PDF) for details. In
    summary, the FAST algorithm deviates slightly from the strict
    definition of simulated annealing, but works well in practice. The SLOW
    algorithm implements strict simulated annealing, but is much slower,
    especially for longer sequences. The FFT algorithm also implements
    strict simulated annealing, and has intermediate speed, but carries
    greater risk of numerical roundoff error. In order to use the FFT
    algorithm, it is necessary to install the FFTW library, and re-compile glam2
    with 'make glam2fft'.

-s
:   Specify the seed for pseudo-random number generation. Change
    this to avoid getting identical results each time the program is run.

## Cosmetic options

-h
:   Show all options and their default settings.

-o
:   Specify an output file name.

-p
:   Print information about the algorithm's state before each
    iteration. The following information is printed: the temperature, the
    number of key positions in the alignment, the number of sequences in
    the alignment, and the alignment's score. In addition, some information
    about each move is printed: for site sampling moves, which sequence is
    picked, and for column sampling moves, which column is picked, whether
    or not it is deleted, and whether the direction is left or right.

-Q
:   Run quietly, suppressing unnecessary messages.

## Warnings

glam2 might occasionally issue this warning: 'accuracy loss due
to numeric underflow'. If this happens, its ability to optimise the
alignment may be harmed: see GLAM2
methods (PDF) for details. To fix this, try increasing -u, or possibly
decreasing -b. Increasing -u somewhat should be harmless, as long as it
stays well below 1.
